# Validation of the French version of the ICHOM adult oral health standard set

**DOI:** 10.1002/cre2.682

**Published:** 2022-11-23

**Authors:** Alba Nicolas‐Boluda, Stephane Simon

**Affiliations:** ^1^ Department of Research Paris France; ^2^ Université de Paris, UFR d'Odontologie Paris France; ^3^ Endo Academie Rouen France

**Keywords:** oral health, patient‐reported outcome measures, quality of life, value‐based health care

## Abstract

**Introduction:**

The ICHOM Adult Oral Health Standard Set (AOHSS) recently developed by the ICHOM Oral Adult Health Working Group is a standard set of outcomes designed for its collection in clinical practice in dental health. The outcome standard set is made up of clinical‐reported outcome measures (CROMs) and patient‐reported outcome measures (PROMs). The purpose of this study was to translate and cross‐culturally adapt the PROM section of the Standard Set in French for France to enable comprehensive evaluation of the patients' oral health quality of life in a French population.

**Methods:**

The questionnaire was translated following the guidelines of the International Society for Pharmacoeconomics and Outcome Research (ISPOR). We included patients consulting in a dentistry clinic (*n* = 127) and seeking dental care. The PROM and CROM data were collected from all patients. Both reliability and the internal consistency were evaluated.

**Results:**

The ICHOM AOHSS was successfully translated into French. We sampled and surveyed 126 patients in a dentistry clinic in France using the French translation of the ICHOM AOHSS. Cronbach's *α* was calculated to measure the internal consistency. The resulting Cronbach's *α* was 0.8, indicating acceptable homogeneity.

**Conclusions:**

The French version of the ICHOM AOHSS shows acceptable psychometric properties in terms of reliability and internal consistency. This translation is suitable for its implementation in a French‐speaking patient population.

## 1 INTRODUCTION

Despite often being neglected, overall oral care and in particular the lack of good oral care leading to oral diseases can have a negative impact in everyday life (Locker & Allen, 2007). Oral diseases can cause considerable pain, alterations in speech and food, and impact on self‐image, self‐esteem, and overall quality of life. One of the ways to improve oral health is measuring its impact on patients. To date, the majority of oral healthcare measures focus on use, access, or process of care (Righolt et al., 2019). For a transition toward a value‐based and patient‐centered care model, healthcare oral providers need to focus more on the overall oral health than the oral disease and for this, there is a need for standardized outcome measurements for oral health. Outcomes from both clinicians' and patients' points of view need to be determined in a simple and reproducible manner.

To measure outcomes from patients' point of view, patient‐reported outcome measures (PROMs) are the most straightforward approach. PROMs are health outcomes that are directly reported by the patients. PROMs are generally determined from questionnaires covering issues and concerns that are specific to the patient; it is a way of obtaining patient‐specific information related to his or her treatment or condition. PROMs allow recording of information related to the patient's quality of life, evolution of certain symptoms, and functional recovery.

In oral health, the patient‐reported assessment is often limited to the evaluation of symptoms using severity scales (Ní Ríordáin et al., 2015). One of the most often used are pain severy scales due to the fact that pain is one of the main symptoms of patients with oral diseases. Currently, no symptom‐specific PROM for oral disease is available; most studies found in the literature report the use of the visual analog scale (VAS) that allows patients to evaluate pain from 1 to 10 (Hawker et al., 2011; Ní Ríordáin et al., 2015). Some studies have also reported the use of PROMs to assess the psychosocial status and the quality of life of patients with oral diseases (Girardi et al., 2011; McCartan, 1995). To date, the few examples of PROMs used to measure outcomes in oral health have been used in the context of clinical trials, not in routine care. To transition toward a value‐based oral health system, generalization of the PROMs used in routine clinical practice is essential.

The International Consortium for Health Outcome Measurements (ICHOM) is a nonprofit organization that aims to accelerate the adoption of value‐based health care around the world. For this purpose, ICHOM develops a standard set of outcomes to be used in routine clinical practice for different diseases and conditions. In the field of oral health, the ICHOM Adult Oral Health Standard Set (AOHSS) has been developed (Ni Riordain et al., 2020). AOHSS is a standard set of outcomes composed of patient‐centered outcomes including clinical‐reported outcome measures (CROMs) and PROMs together with a series of case‐mix variables. The case‐mix variables are patient characteristics and risk factors that will affect the patient‐centered outcomes. The AOHSS represents a minimum set of outcomes to be collected in routine clinical practice, research, and population health. The AOHSS has been developed by the ICHOM Oral Adult Health Working Group, which includes clinicians, patient advocates, researchers, and public health experts in dental health. The main clinical practice in which it has been used are routine check‐ups, caries, and periodontal disease. The AOHSS is made up of 31 outcomes that include a total of 80 measures: 25 PROM and 55 CROM. The 25 PROMs include questions regarding the impact of patients' oral health on their oral function and a record of pain and oral hygiene practices. The CROMs include medical history, a record of caries and of periodontal disease, and the different types of dental treatments provided (Ni Riordain et al., 2020).

As proven in case studies in other fields of medicine, the use of these outcome standard sets in routine clinical practice can have a major impact toward the improvement of quality of care. Improvements could include higher relevancy in proposed care, decrease of delays in clinical practice, and overall care more focused on what is important for patients (ICHOM, 2021). With the aim of implementing this standard set in dentistry clinics in France, the purpose of this study was to translate and culturally adapt the PROM section (the 25 patient‐reported outcomes) of the ICHOM AOHSS into French and assess its psychometric properties.

## 2 MATERIALS AND METHODS

### 2.1 Study population

We performed an observational study between March 22, 2021 and April 23, 2021 at the Cabinet Dentaire Val de Fontenay (France).

### 2.2 Sample size

Sample size was calculated according to the recommendations of Hatcher et O'Rourke (Hatcher & O'Rourke, 2014): the sample size needs to be at least five times the number of items in the questionnaire, with the inclusion of at least 100 patients. The AOHSS has 13 items, this means that at least 100 patients are needed for the study. Considering a dropout rate of around 30%, 130 patients were needed to validate the translation of the AOHSS.

### 2.3 Translation

**Figure 1:**
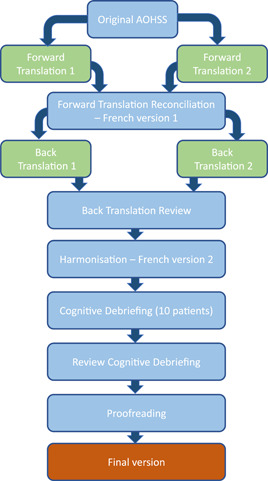
Protocol followed for the translation of the ICHOM AOHSS. AOHSS, Adult Oral Health Standard Set

Translation and cross‐cultural adaptation were performed following the guidelines for the translation and cultural adaptation process of the International Society of Pharmacoeconomics and Outcomes Research (ISPOR) for patient‐reported outcomes (Wild et al., 2005). The process of the French translation and validation of the ICHOM AOHSS is outlined in Figure 1.

#### 2.3.1 Forward translation

The forward translation from English to French was performed by two independent native qualified French translators who were unfamiliar with the ICHOM AOHSS. Two independent French versions of the questionnaire were developed; translations were then compared and combined into a single version (version 1).

#### 2.3.2 Back‐translation and back‐translation review

The reconciled French version of the AOHHS (version 1) was then back‐translated into English by two independent native English translators who were proficient in French. The back‐translation was reviewed and compared to the original version of the questionnaire in terms of identical concepts. A new revised version (version 2) of the French questionnaire was developed and was then ready to be pilot‐tested on a sample of patients.

#### 2.3.3 Cognitive debriefing

The objective of the study was to confirm that the translation was accurately understood. A pilot test was performed on a sample of 10 volunteers who were patients of the dentistry clinic where this study was performed. The inclusion criteria for the patients were as follows: native French speakers, older than 18 years of age, and willing to participate in the study. All patients received detailed information on the study and were asked to provide informed consent.

A face‐to‐face semi‐structured interview was conducted to determine how well the questions were understood. The aim was to identify any semantic or conceptual deviations (Hall et al., 2018). Patients were asked to complete the translated questionnaire on their own. Then, the questionnaire was read out loud by the researchers while the patients read the questionnaire. Next, the researcher asked more specific questions focused on (i) identifying difficult words or phrases, (ii) explaining the questions in their own words, and (iii) suggesting alternative wording.

#### 2.3.4 Review and finalization of the translation

After reviewing the cognitive debriefing, a final version of the questionnaire was proposed.

### 2.4 Data collection

Once the final version of the French translation of the ICHOM AOHSS was ready, patients attending routine visits for dental care in a dentistry clinic in France (Cabinet Dentaire Val de Fontenay) were invited to complete the PROM questionnaire before their consultation. Patients were recruited between March and April 2021. Patients who had difficulty in understanding French, were under guardianship or curatorship, and were underage (younger than 18 years of age) were excluded from the study.

In addition to the PROM data, the following data were collected: age, gender, educational level, type of treatment received, sugar consumption, tobacco use, alcohol consumption, and financial status. The different types of treatments were classified into three types: prevention or control (i.e., fluoride varnish, oral hygiene instructions, sealants, deep scaling, etc.); preservation (i.e., periodontal surgery, fillings, crowns, etc.), and extraction.

The questionnaires were administered using pencil and paper. Detailed information about the questionnaire was provided to the patients before they completed the questionnaire; only patients who confirmed that they were not opposed to the use of the data collected through the questionnaire in a scientific study were given the questionnaire.

### 2.5 Statistical analysis

The results are presented as mean with standard deviation (SD).

Reliability measures the degree to which an instrument is free of random error (Aaronson et al., 2002). To measure reliability, internal consistency was measured. Internal consistency evaluates the extent to which question items within the same construct measure the same conceptual domain and determines whether it is valid to sum the item score (Tavakol & Dennick, 2011). Internal consistency was statistically evaluated using Cronbach's *α* coefficient. The threshold for Cronbach's *α* to be considered as having an acceptable internal consistency is >0.7. The internal consistency for each question (Cronbach's *α* is item deleted) was also assessed for each item.

All statistical analyses were performed using R Studio and Microsoft Excel.

This study was approved by the Ethics Committee *Comité de Protection des Personnes Ile de France II* (N°ID RCB 202‐A03258‐31, N° projet 20.12.14.68534 RIPH3 HPS).

## 3 RESULTS

One hundred and thirty‐three participants were invited to participate in this study; of these, 126 (94.7%) patients agreed to participate. The mean age of the participants was 41.7 years (SD 12.6), with a higher percentage of male participants (61.9%), and most patients had completed lower secondary education (26.9%). All demographic data are summarized in Table 1. The main treatment received during their visit was prevention or control (45.2%), preservation (41.3%), and extraction (13.5%).

**Table 1:** Participants' demographic data

	Total
Number of patients, *n* (%)	126 (100%)
*Demographic data*	
Gender	‐
Male	78 (61.9)
Female	48 (38.1)
Mean age (SD)	41.79 (13.59)
Min–max	18–81
Educational level	
Lower secondary education	34 (26.9)
Upper secondary education	31 (24.6)
Short‐cycle tertiary education	22 (17.4)
Bachelor's degree or equivalent	14 (11.1)
Master's degree or equivalent	22 (17.4)
Doctoral degree or equivalent	3 (2.3)
Type of treatment	
Prevention or control	57 (45.2)
Preservation	52 (41.3)
Extraction	17 (13.5)

Abbreviation: SD, standard deviation.

All patients completed the questionnaire, with no unanswered questions that could generate unknown items. The distribution of the responses to the French version of the ICHOM AOHSS is shown in Table 2, and the descriptive distribution is summarized in Table 3. The floor effect was negligible for each item.

**Table 2:** Distribution of the responses to the French version of the ICHOM AOHSS

	*n* (%)				
	Very poor *Très mauvais*	Poor *Mauvais*	Fair *Satisfaisant*	Good *Bon*	Very good *Très bon*
How is the health of your mouth, teeth, and gums?	0 (0)	13 (10.3)	45 (35.7)	52 (41.2)	16 (12.7)
*Quel est l'état de santé de votre bouche, de vos dents et de vos gencives?*					

	Never *Jamais*	Hardly ever *Presque jamais*	Sometimes *Quelquefois*	Fairly often *Assez souvent*	Very often *Très souvent*
In the last 6 months, how often have you felt embarrassed or self‐conscious because of problems with your teeth, gums, or dentures?	4 (3.2)	9 (7.2)	46 (36.5)	30 (23.8)	37 (29.4)
*Au cours des six derniers mois, vous êtes‐vous senti(e) embarrassé(e) ou gêné(e) en raison de problèmes de dents, gencives ou de prothèses?*					
In the last 6 months, how often have you found it hard to interact with others because of problems with your teeth, gums, or dentures?	0 (0)	5 (4.1)	26 (20.7)	30 (23.8)	65 (51.6)
*Au cours des six derniers mois, vous a‐t‐il été difficile d'interagir avec d'autres en raison de problèmes de dents, gencives ou prothèses?*					
In the last 6 months, how often have you found it hard to carry out your usual work activities or responsibilities because of problems with your teeth, gums, or dentures? This includes at your job or in your home.	0 (0)	2 (1.6)	16 (12.7)	25 (19.8)	83 (65.8)
*Au cours des six derniers mois, vous a‐t‐il été difficile de mener vos activités ou responsabilités professionnelles habituelles en raison de problèmes de dents, gencives ou prothèses? Ceci concerne à la fois votre travail et votre domicile*					
In the last 6 months, how often have you felt embarrassed smiling, laughing, and showing your teeth because of problems with your teeth, gums, or dentures?	7 (5.5)	6 (4.7)	20 (15.8)	27 (21.4)	66 (52.3)
*Au cours de six derniers mois, vous êtes‐vous senti(e) embarrassé(e) de sourire, rire et montrer vos dents en raison de problèmes de dents, gencives ou prothèses?*					
In the last 6 months, how often have you been happy with the way your teeth, gums, or dentures look?	4 (3.7)	25 (19.8)	31(24.6)	37 (29.4)	29 (23)
*Au cours de six derniers mois, avez‐vous été content(e) de l'aspect de vos dents, gencives ou prothèses?*					
In the last 6 months, how often have you found it hard to eat because of problems with your teeth, gums, or dentures?	9 (7.1)	11 (8.7)	42 (33.3)	29 (23)	35 (27.7)
*Au cours de six derniers mois, vous a‐t‐il été difficile de manger ou boire à cause de vos problèmes de dents, gencives ou prothèses?*					
In the last 6 months, how often have you had to change what you eat or drink because of problems with your teeth, gums, or dentures?	7 (5.5)	10 (7.9)	26 (20.6)	30 (23.8)	53 (42)
*Au cours de six derniers mois, avez‐vous dû changer ce que vous mangez ou buvez à cause de vos problèmes de dents, gencives ou prothèses?*					
In the last 6 months, how often have you found it hard to speak clearly because of problems with your teeth, gums, or dentures?	1 (0.8)	4 (3.2)	10 (7.9)	33 (26.1)	78 (61.9)
*Au cours de six derniers mois, avez‐vous eu des difficultés à parler clairement en raison de problèmes de dents, gencives ou prothèses?*					
In the last 6 months, how often have you had trouble sleeping because of problems with your teeth, gums or dentures?	6 (4.7)	3 (2.4)	13 (10.3)	35 (27.7)	69 (54.7)
*Au cours de six derniers mois, avez‐vous eu des problèmes pour dormir en raison de problèmes de dents, gencives ou prothèses?*					
In the last 6 months, how often have you had pain in your mouth?	8 (6.3)	9 (7.1)	54 (42.8)	21 (16.6)	34 (26.9)
*Au cours de six derniers mois, avez‐vous eu des douleurs dans la bouche?*					
In the last 6 months, how often has your mouth felt dry?	3 (2.3)	9 (7.1)	35 (27.7)	42 (33.3)	36 (28.6)
*Au cours de six derniers mois, avez‐vous eu la bouche sèche?*					
In the last 6 months, how often have your teeth been sensitive to hot or cold food or drinks?	7 (5.5)	13 (10.3)	49 (38.8)	37 (29.4)	20 (15.8)
*Au cours de six derniers mois, vos dents ont‐elles été sensibles aux aliments ou boissons chaudes ou froides?*					

Abbreviation: AOHSS, Adult Oral Health Standard Set.

**Table 3:** Descriptive distribution of the French version of the ICHOM AOHSS

	Percentage of items not answered	Theoretical range	Observed range	Floor effects (% score = 0)	Mean (SD)	Median (IQR)
General health	0	0–4	1–4	0	2.56 (0.8)	1 (1)
Self‐confidence	0	0–4	0–4	3.17	2.69 (1.1)	2.5 (2)
Social participation	0	0–4	1–4	0	3.21 (0.9)	3.5 (1)
Productivity	0	0–4	1–4	0	3.50 (0.7)	4 (1)
Smiling	0	0–4	0–4	5.55	3.11 (1.2)	3 (2)
Aesthetic satisfaction	0	0–4	0–4	3.17	2.49 (1.1)	2 (1)
Ability to eat	0	0–4	0–4	7.14	2.55 (1.2)	2.5 (2)
Food alteration	0	0–4	0–4	5.55	2.88 (1.2)	2.5 (2)
Ability to speak	0	0–4	0–4	0.79	3.45 (0.8)	3.5 (1)
Ability to sleep	0	0–4	0–4	4.76	3.25 (1.1)	4 (1)
Oral pain	0	0–4	0–4	6.34	2.51 (1.2)	3 (2)
Dry mouth experience	0	0–4	0–4	2.38	2.81 (1.2)	1.5 (2)
Sensitivity experience	0	0–4	0–4	5.55	2.39 (1.5)	2 (1)

Abbreviations: AOHSS, Adult Oral Health Standard Set; SD, standard deviation.

Cronbach's *α* coefficient, average inter‐item correlation, mean, and summary scores with SDs of the French version of the ICHOM AOHSS are presented in Table 4.

**Table 4:** Cronbach's *α* coefficient, average inter‐item correlation, mean, and summary scores with SDs

Questionnaire	*n*	Cronbach's	Average inter‐item correlation	Mean summary score	SD (mean summary score)
*ICHOM AOHSS FR*	126	0.81	0.55	37.44	7.47

Abbreviations: *N*, number of participants; SD, standard deviation.

Table 5 presents the mean scores of each item with SDs, corrected item–total correlation, and Cronbach's *α* when one item was deleted. All item–total correlations were above 0.2. Even if one item was deleted, Cronbach's *α* coefficient remained higher than 0.7.

**Table 5:** Mean item scores, SDs, corrected item–total correlations, and Cronbach's *α* coefficient when the item was deleted of the French version of the ICHOM AOHSS

	Mean	SD	Item–total correlation	Cronbach's *α* if item deleted
General health	2.56	0.84	0.34	0.81
Self‐confidence	2.69	1.07	0.59	0.79
Social participation	3.23	0.91	0.58	0.79
Productivity	3.51	0.77	0.56	0.79
Smiling	3.11	1.17	0.53	0.80
Esthetic satisfaction	2.49	1.14	0.22	0.82
Ability to eat	2.55	1.19	0.78	0.77
Food Alteration	2.88	1.21	0.73	0.77
Ability to speak	3.45	0.83	0.62	0.79
Ability to sleep	3.25	1.05	0.64	0.78
Oral pain	2.51	1.15	0.66	0.78
Dry mouth experience	2.81	1.03	0.31	0.81
Sensitivity experience	2.39	1.05	0.62	0.78

Abbreviations: AOHSS, Adult Oral Health Standard Set; SD, standard deviation.

## 4 DISCUSSION

The purpose of this study was to translate the ICHOM AOHSS into French. The translation approach used ensured that the PROMs were culturally and linguistically appropriate. When translating existing questionnaires for a different country, and therefore culture, the process must not only take into consideration the language but also the cultural context (Epstein et al., 2015). To ensure that the translation of the ICHOM AOHSS accounted for cross‐cultural adaptations, the guidelines of the International Society for Pharmacoeconomics and Outcome Research (ISPOR) were followed. These guidelines have also been used in other PROM translation and validation studies (Huang et al., 2021; Katz et al., 2021; Lorenzen et al., 2018).

In this study, we present the validation of the French version of ICHOM AOHSS; we prove that it is equivalent to the English version (Ni Riordain et al., 2020) and comprehensible by French‐speaking patients. The Cronbach *α* coefficient was congruent and indicated adequate reliability for all the items.

In daily clinical practice, there is a lack of evidence of the quality of oral health in patients. The ICHOM AOHSS is the first patient‐reported outcome standard set proposed for general oral health. Determination of PROMs in oral health will allow shifting the organization of oral care toward addressing the concerns and the priorities defined by patients (Dawson et al., 2010). The use of  PROMs in routine practice is still not common for oral health (Rosen et al., 2016) and it is true that there are major challenges linked to their use and interpretation in routine clinical practice (Tsakos et al., 2012). The information from AOHSS can be very valuable during patient consultations, as it can be used by both clinicians and patients as a shared decision‐making tool to develop an adapted oral care pathway for the patient.

This study has shown that this version of AOHSS in French demonstrated robust psychometric properties. It is therefore suitable for use in French patients in dentistry clinics for everyday oral care. Applicability in daily clinical practice can be improved by using a digital data collection system instead of classical paper and pen, as was the case in this study. The digital format can indeed improve data quality and facilitate real‐time use of these data during consultations. Further work is therefore needed to demonstrate the feasibility and validity of a digital version of AOHSS in French.

## 5 CONCLUSIONS

In this study, a French version of the PROM section of the ICHOM AOHSS has been developed. This version was evaluated and proven to have good psychometric properties. This version is suitable for French‐speaking patients.

## AUTHOR CONTRIBUTIONS

**Alba Nicolas‐Boluda** was involved in the conceptualization, methodology, and investigation of the study, and writing of the manuscript – original draft preparation. **Stephane Simon** was involved in the methodology and investigation of the study, and writing of the manuscript – review and editing. All authors have read and agreed to the published version of the manuscript.

## CONFLICT OF INTEREST

The authors declare no conflict of interest.

## Data Availability

The authors confirm that the data supporting the findings of this study are available within the article or its supplementary materials.
